# The Impact of Genetic Variation on Duck Hepatitis A Virus (DHAV) Vaccine Efficacy: A Comparative Study of DHAV-1 and DHAV-3 Against Emerging Variant Strains

**DOI:** 10.3390/vaccines12121416

**Published:** 2024-12-16

**Authors:** Sang-Won Kim, Cheng-Dong Yu, Jong-Yeol Park, Xiu-Li Ma, Tong Zhu, Yu-Feng Li, Se-Yeoun Cha, Hyung-Kwan Jang, Min Kang, Bai Wei

**Affiliations:** 1 Department of Avian Diseases, College of Veterinary Medicine and Center for Avian Disease, Jeonbuk National University, Iksan 54596, Republic of Korea; sangwonkim@jbnu.ac.kr (S.-W.K.); jyp0410@jbnu.ac.kr (J.-Y.P.); hkjang@jbnu.ac.kr (H.-K.J.); 2 Shandong Provincial Key Laboratory of Livestock and Poultry Breeding, Institute of Poultry Science, Shandong Academy of Agricultural Sciences, Jinan 250100, China; 3 Bio Disease Control (BIOD) Co., Ltd., Iksan 54596, Republic of Korea

**Keywords:** duck hepatitis A virus, genetic diversity, vaccine efficacy, cross-protection

## Abstract

**Background/Objective:** Duck virus hepatitis (DVH), caused by duck hepatitis A virus (DHAV), poses significant challenges to duck farming due to high mortality rates in young ducklings. Despite the widespread use of live attenuated vaccines, the genetic diversity within DHAV strains has diminished their cross-protection efficacy. This study aimed to evaluate the cross-protective efficacy of current DHAV-1 and DHAV-3 vaccines against genetically divergent wild strains. **Methods:** Phylogenetic analyses of the VP1 genes from DHAV-1 and DHAV-3 were conducted. Both DHAV-1 and DHAV-3 vaccines were tested in ducklings, with and without maternal-derived antibodies (MDA), through challenge trials with homologous and heterologous strains. **Results:** In the phylogenetic analysis, compared to vaccine strains, DHAV-1 and DHAV-3 field variant strains were classified into different genotypes. In ducklings without MDA, the DHAV-1 vaccine provided 60% survival against homologous strains by 2 days post-vaccination (DPV) and complete protection by 4 DPV, while survival rates against heterologous strains ranged from 40 to 60%. In ducklings with MDA, the DHAV-1 vaccine provided full protection with an additional vaccination for day-old ducklings against heterologous strains. The DHAV-3 vaccine conferred complete protection against both homologous and heterologous strains by 2 DPV, regardless of MDA presence. **Conclusions:** The DHAV-3 vaccine demonstrated robust cross-protection across genotypes, while the DHAV-1 vaccine showed limitations against genetically divergent strains. These findings highlight the necessity for genotype-matched vaccines and optimized immunization strategies to enhance protection against evolving DHAV field strains.

## 1. Introduction

Duck virus hepatitis (DVH) is a notifiable and World Organization for Animal Health (WOAH)-listed disease that is contagious, lethal, and economically devastating to duck-raising industries [1,2]. Affected birds, primarily young ducklings, exhibit various nervous symptoms and, upon postmortem examination, display hepatitis, enlarged hemorrhagic livers, an enlarged spleen, and swollen kidneys, typically associated with high mortality [1]. Since its discovery in 1945 in the United States, DVH has spread significantly, worldwide [3]. Vaccination has been the primary strategy for preventing DHAV infection; both live attenuated and inactivated oil emulsion vaccines have been effectively used for decades to control the disease [4]. Moreover, current DHAV vaccination strategy in ducklings was divided into two categories. First, similar to vaccine strategy in other poultry diseases such as Newcastle disease (ND), infectious bursal disease (IBD), and avian influenza, the vaccination of breeder ducks for inducing maternal-derived antibodies (MDAs) against DHAV in progeny [5,6]. MDAs can be transferred from vaccinated breeder ducks to their offspring and have a critical role in providing essential passive immunity against DHAV in early-life stages of ducklings [4]. Second, enhancing vaccine efficacy in progeny by live attenuated vaccine. Similar to IBD vaccination in chickens, priming live attenuated vaccines can interfere with pre-existing MDAs [7]; however, this strategy can stimulate a strong immune response in the same way as a boosting vaccination or natural infections. For these reasons, priming vaccination was accessed in DHAV vaccine and other poultry diseases such as ND and infectious bronchitis (IB) [8,9].

The causative agent of DVH, duck hepatitis A virus (DHAV), is a positive-sense, single-stranded RNA virus. Its genome contains a single open reading frame (ORF) that encodes a polyprotein, which is processed into the major capsid proteins VP0, VP3, and VP1 [10,11]. Notably, VP1 is the primary structural capsid protein and plays a critical role in viral antigenicity, immunogenicity, receptor binding, and virulence. As such, it is crucial for the genotyping and classification of DHAV strains [3,12]. DHAVs are classified into three distinct serotypes, based on VP1 sequence analysis and virus neutralizing capacity: DHAV-1, DHAV-2, and DHAV-3 [3,13]. Moreover, DHAV-1 strains have been classified into four main genotypes (groups 1 to 4), with group 1 further subdivided into three subgroups (1.1, 1.2, and 1.3). Simultaneously, DHAV-3 strains are categorized into three primary groups (groups 1 to 3) [14]. This genetic diversity within and between serotypes significantly impacts viral antigenicity, diminishing the cross-protective efficacy between vaccine strains and genetically divergent field strains; no cross-protection has been observed between DHAV-1 and DHAV-3 [4,14]. Recent studies have documented the emergence of new genotypes and subgenotypes within DHAV-1 and DHAV-3, with vaccinated flocks still experiencing outbreaks and high mortality rates, highlighting a significant challenge in effective disease control [15,16]. Given these observations, the potential for vaccine strain mismatch and the resulting diminished cross-protection highlights the urgency of assessing the efficacy of current DHAV vaccines against genetically distinct field strains. However, many DHAV vaccine studies were focused on vaccine efficacy against homologous field DHAV-1 and DHAV-3 strains [17,18], and cross-protection efficacy of MDA and priming of live attenuated vaccine in progeny ducklings against variant field strains were limited.

A comprehensive understanding of cross-protection limitations is essential for informing targeted advances in vaccine design and achieving broad-spectrum immunity against diverse DHAV strains. Consequently, this study aims to evaluate the cross-protective efficacy of existing DHAV-1 and DHAV-3 vaccines, with a focus on their ability to protect against genetically diverse wild strains. By elucidating the limitations and potential of current vaccines, this research intends to provide insights into strategies for optimizing DHAV control and enhancing the resilience of duck flocks against circulating and emerging DHAV genotypes.

## 2. Materials and Methods

### 2.1. Viruses and Vaccines

The virulent DHAV-1 strain DRL-62 was obtained from the American Type Culture Collection (Manassas, VA, USA), and the virulent DHAV-3 strain AP-04203 was sourced from the Animal and Plant Quarantine Agency (Gimcheon, Republic of Korea). The Korean live attenuated DHAV-1 vaccine strain HSBP100, and DHAV-3 vaccine strain AP-04203P100, which were previously described in the literature [17,18] were utilized in this study. Chinese variant field strains, DHAV-1 (D-20220155 and D-20220906) and DHAV-3 (D-20220615 and D-202210113), were isolated from liver samples of ducklings exhibiting neurological signs and high mortality. The viral strains were propagated in the allantoic cavities of 8-day-old embryonated specific-pathogen-free (SPF) chicken eggs (Hy-Vac Laboratory, Redfield, IA, USA) at 37 °C for 5 days, passaged twice, and preserved at −70 °C. The virus infectious titer for each virus strains were determined by 50% embryo lethal dose (ELD_50_) in embryonated SPF chicken eggs, and ELD_50_ was calculated by using the Reed and Muench method [19].

### 2.2. Sequence Analysis of DHAV VP1

Viral RNA was extracted using the MagMAX™-96 AI/ND Viral RNA Isolation Kit (Thermo Fisher Scientific, Vilnius, Lithuania) and the KingFisher Duo Prime Purification System (Thermo Fisher Scientific, Waltham, MA, USA), according to the manufacturer’s instructions. Complementary DNA (cDNA) was synthesized from this viral RNA by reverse transcription (RT). In the RT steps, 1 µL of random primer (9-mers; TaKaRa Bio Inc., Kusatsu, Japan) was added to every 10 µL of extracted RNA, heated at 70 °C for 5 min, followed by cooling in an ice bath for 5 min. Subsequently, a mixture was prepared to contain 4 µL of GoScript™ 5X RT reaction buffer (Promega, Madison, WI, USA), 1 µL of 10 mM dNTP, 1.5 µL of 25 mM MgCl_2_ (Promega, Charbonnie, France), 0.5 µL of 40 units Recombinant RNasin^®^ Ribonuclease Inhibitor (Promega, Madison, WI, USA), 1 µL of GoScript™ reverse transcriptase (RTase, Promega, Madison, WI, USA), and 1 µL of diethylpyrocarbonate-treated water (DEPC water, Biosesang, Yongin, Republic of Korea). The RT reaction, totaling 20 µL, was incubated at 25 °C for 5 min, followed by 42 °C for 1 h. Finally, to deactivate the RTase, it underwent a 70 °C incubation for 15 min. After RT steps, synthesized cDNA was subject to PCR. For the amplification of the full-length VP1 gene from DHAV-1 and DHAV-3, primer sets were adopted from previous studies [20,21].

Positive PCR products were verified with 1.5% agarose gel electrophoresis. The PCR products of the expected lengths were purified with the QIAquick gel extraction kit (Qiagen, Chatsworth, CA, USA), and sequenced at Bioneer Corporation (Daejeon, Republic of Korea). The phylogenetic analysis of the VP1 gene was carried out using MEGA 7.0 with the neighbor-joining method (1000 bootstrap replicates) and the maximum composite likelihood model. Sequence alignments and pairwise sequence comparisons within the VP1 regions of DHAV-1 and DHAV-3 were assessed using DNAMAN version 6.0 (Lynnon, Vaureuil, QC, Canada).

### 2.3. Virus Neutralization Assay

For the determination of virus-neutralizing (VN) antibody titer of MDA in used 1-day-old ducklings in animal experiments and the evaluation of virus cross-neutralization assay between different DHAV-1 and DHAV-3 strains, VN assays were conducted on the embryonated SPF chicken eggs. The VN assay involved constant amounts of viruses and diluted serum. Briefly, 2-fold serial dilutions of inactivated hyperimmune chicken sera were mixed with 200 ELD_50_ of virus for 60 min at 37 °C. The hyperimmune chicken sera used against DHAV-1 and DHAV-3 in this study were previously outlined [22]. Subsequently, the serum–virus mixtures were inoculated into 9-day-old embryonated SPF chicken eggs via the allantoic route. Controls included sera only and viruses only. Embryonic eggs were monitored daily, and dead embryos were noted up to 6 days post-inoculation. Serum neutralizing titers were determined by the minimal serum dilution that prevented chicken embryo death, with mean values obtained from two repeated experiments [21].

### 2.4. Vaccination and Challenge Experiment

All experimental and animal management procedures were undertaken to ensure animal health and well-being throughout the study. Additionally, the study was approved by, and in accordance with, the requirements of the Animal Care and Ethics Committee of Jeonbuk National University. The animal facility at Jeonbuk National University is fully accredited by the National Association of Laboratory Animal Care (Approval number: NON2023-009) and all used ducklings were kept in temperature-controlled isolators and provided ad libitum access to feed and water.

In experiments 1 and 2, 1-day-old white Pekin ducklings, obtained from a commercial hatchery, underwent screening to ensure they were free of DHAV infection using virus neutralization testing and quantitative real-time polymerase chain reaction (qRT-PCR) [23,24].

In experiment 1, the protective efficacy of DHAV-1 vaccine in ducklings without MDA was accessed. For this experiment, one hundred 1-day-old ducklings were divided into five groups of twenty ducklings. Forty ducklings in two groups (G2 and G4) were vaccinated with single dose of DHAV-1 vaccine strain DHV-HSBP100 intramuscularly (IM), and sixty ducklings in three groups (G1, G3 and G5) were inoculated with PBS by the IM route. At 2 and 4 days post-vaccination (DPV), ten ducklings in G1 and G2 were challenged with 10^3.0^ ELD_50_/0.2 mL of virulent DHAV-1 strain DRL-62 by the IM route, and ten ducklings in G3 and G4 were challenged with 10^3.0^ ELD_50_/0.2 mL of DHAV-1 variant field strain D-220906 by the IM route, respectively. Twenty ducklings in G5 were not challenged, and remained as a negative control. In the virus challenge group (G1–4), duckling survival was monitored for 7 days following the challenge.

In experiment 2, the protective efficacy of the DHAV-3 vaccine in ducklings without MDA was accessed. For this experiment, one hundred 1-day-old ducklings were divided into five groups of twenty ducklings. Forty ducklings in two groups (G2 and G4) were vaccinated with a single dose of DHAV-3 vaccine strain AP-04203P100 by the IM route, and sixty ducklings in three groups (G1, G3 and G5) were inoculated with PBS by the IM route. At 2 and 4 DPV, ten ducklings in G1 and G2 were challenged with 10^4.0^ ELD_50_/0.2 mL of DHAV-3 virulent strain AP-04023 by the IM route, and ten ducklings in G3 and G4 were challenged with 10^4.0^ ELD_50_/0.2 mL of DHAV-3 variant field strain D-220615 by the IM route, respectively. Twenty ducklings in G5 were not challenged, and remained as a negative control. In the virus challenge group (G1–4), duckling survival was monitored for 7 days following the challenge.

In experiments 3 and 4, 1-day-old white Pekin ducklings derived from breeder ducks previously immunized with a bivalent DHAV vaccine (DHV-HSBP100 and AP-04203P100) were utilized. The ducklings were assessed using qRT-PCR, and serum samples were collected to verify the VN titer.

In experiment 3, the protective efficacy of the DHAV-1 vaccine in ducklings with MDA was accessed. For this experiment, fifty 1-day-old ducklings were divided into five groups of ten ducklings. Twenty ducklings in two groups (G2 and G4) were vaccinated with a single dose of DHAV-1 vaccine strain DHV-HSBP100 by the IM route, and thirty ducklings in three groups (G1, G3 and G5) were inoculated with PBS by the IM route. At 2 DPV, ten ducklings in G1 and G2 were challenged with 10^3.0^ ELD_50_/0.2 mL of DHAV-1 virulent strain DRL-62 by the IM route, and ten ducklings in G3 and G4 were challenged with 10^3.0^ ELD_50_/0.2 mL of DHAV-1 variant field strain D-220906 by the IM route. Ten ducklings in G5 were not challenged, and remained as a negative control. Duckling survival was monitored for 7 days following the challenge.

In experiment 4, the protective efficacy of the DHAV-3 vaccine in ducklings with MDA was accessed. For this experiment, fifty 1-day-old ducklings were divided into five groups of ten ducklings. Twenty ducklings in two groups (G2 and G4) were vaccinated with single dose of DHAV-3 vaccine strain AP-04203P100 by the IM route, and thirty ducklings in three groups (G1, G3 and G5) were inoculated with PBS by the IM route. At 2 DPV, ten ducklings in G1 and G2 were challenged with 10^4.0^ ELD_50_/0.2 mL of DHAV-3 virulent strain AP-04023, and ten ducklings in G3 and G4 were challenged with 10^4.0^ ELD_50_/0.2 mL of DHAV-3 variant field strain D-220615 by the IM route. Ten ducklings in G5 were not challenged, and remained as a negative control. Duckling survival was monitored for 7 days following the challenge.

## 3. Results

### 3.1. Genetic and Phylogenetic Analysis

**Figure 1:**
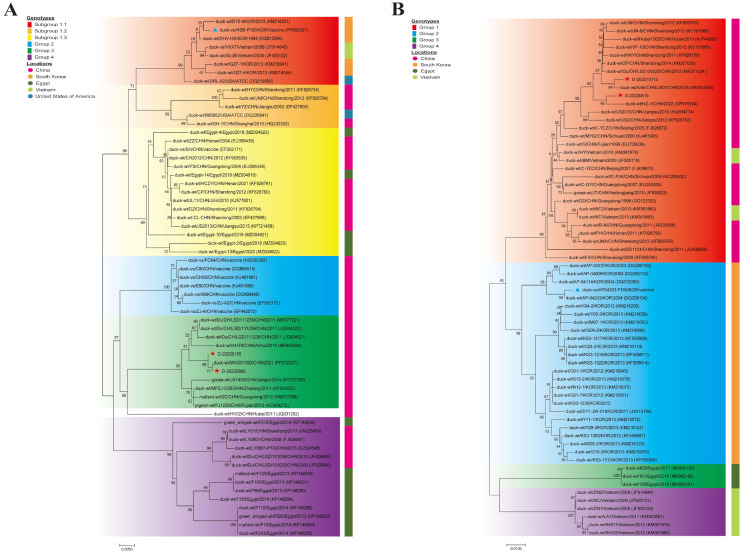
Phylogenetic tree of DHAV-1 (**A**) and DHAV-3 (**B**) VP1 gene nucleotide sequences. Phylogenetic analysis of the VP1 gene was conducted using MEGA 7.0 with the maximum composite likelihood model and 1000 bootstrap replicates. The scale bar indicates the phylogenetic distance, representing 0.05 substitutions per nucleotide site. Vaccine and wild strains used in this study are indicated by blue triangles (vaccine strains) and red asterisks (variant field strains).

For the phylogenetic analysis, VP1 genes for DHAV-1 were classified into four groups (groups 1–4), and intra-group nucleotide sequence identities showed group 1 (93.8%–100%), group 2 (98.9%–99.9%), group 3 (97.3%–100%), and group 4 (96.1%–100%), respectively (Figure 1A); group 1 was further subdivided into three subgroups (1.1, 1.2, and 1.3). In group 1, the DHAV-1 vaccine strain HSBP100 and DHAV-1 virulent strain DRL-62 were clustering within subgroup 1.1. Variant field strains D-20220155 and D-20220906 were clustered closely with previously identified strains in group 3. For DHAV-3, strains were grouped into four main clusters (Group 1–4) with the mean intra-group nucleotide sequence identities of group 1 (93.6%–100%), group 2 (94.6%–100%), group 3 (99.7%–99.9%), and group 4 (99.3%–100%) (Figure 1B). DHAV-3 vaccine strains AP-04203P100 and their parental virulent strain AP-04203 were clustered closely with previously identified strains in group 2, while variant field strains D-20220155 and D-202210113 were clustered within group 1.

**Figure 2:**
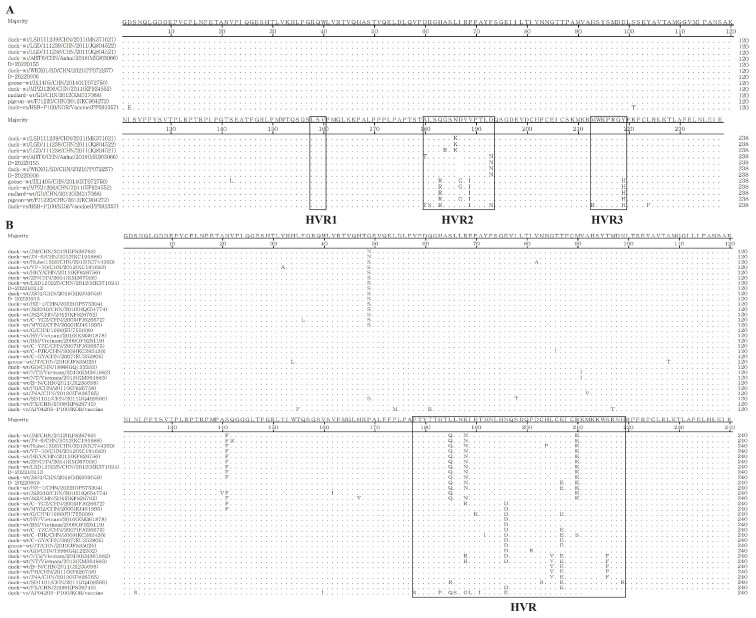
Amino acid comparison of the VP1 gene sequences of DHAV-1 (**A**) and DHAV-3 (**B**), including the hypervariable regions (HVRs) in both DHAV-1 and DHAV-3.

The amino acid alignment for DHAV-1 and DHAV-3 with the vaccine strain and variant field strains are shown in Figure 2. For the amino acid analysis of DHAV-1, compared with the DHAV-1 vaccine strain HSBP100, DHAV-1 variant field strain D-20220906 revealed eight deduced amino acid substitutions (T101S, T180A, S181L, R183Q, I189V, R213M, H219Y, and F224L) and a deletion at position 187, predominantly within hypervariable regions HVR-2 (180–193) and HVR-3 (213–219) (Figure 2A). For DHAV-3 (Figure 2B), 16 deduced amino acid substitutions (F35L, G49S, M54L, R61Q, T97M, S123N, I160V, S178P, P183H, S186L, G188N, L189I, I191T, E196N, K207E, and R210K) were found in DHAV-3 variant field strain D-20220615, compared with DHAV-3 vaccine strain AP-04203P100, and 9 substitutions were within the C-terminus of HVR (178–219).

### 3.2. Cross-Neutralization Between DHAV Strains

Cross-neutralization results demonstrated that within-group DHAV-1 variant field strains (D-20220155 and D-20220906, group 3) exhibited high cross-neutralizing activity. However, these group 3 strains displayed low cross-neutralizing titers against DHAV-1 virulent strain DRL-62 (group 1.1) (Table 1). For DHAV-3, Chinese variant field strains (D-20220615 and D-202210113, group 1), and virulent DHAV-3 strain AP-04203 (group 2) showed high cross-neutralizing activity both within and between groups 1 and 2 (Table 2).

### 3.3. Protective Efficacy of DHAV-1 Vaccine (Group 1.1)

The efficacy of the DHAV-1 vaccine strain HSBP100 (group 1.1) was assessed in two experimental setups (experiment 1 and 3) to determine its protective effect against heterologous DHAV-1 strains. In the first experiment (experiment 1), ducklings lacking MDA were vaccinated and subsequently challenged at 2 and 4 DPV with the homologous DHAV-1 virulent strain DRL-62 (group 1.1) or the heterologous DHAV-1 variant field strain D-20220906 (group 3). Against the homologous DHAV-1 strain DRL-62 (group 1.1), survival was recorded at 60% by 2 DPV, reaching full protection by 4 DPV; none of the surviving ducklings exhibited clinical symptoms throughout the study period (Table 3). In contrast, for the heterologous DHAV-1 strain D-20220906 (group 3), survival rates were 40% at 2 DPV, improving to 60% by 4 DPV.

In the second experiment (experiment 3), the protective efficacy of the DHAV-1 vaccine strain HSBP100 (group 1.1) in ducklings with MDA was assessed. Antibody titers against the homologous DHAV-1 virulent strain DRL-62 (group 1.1) in 1-day-old ducklings were recorded at 3.4 ± 0.4 log2 via virus-neutralization tests. Ducklings with MDA were challenged with both homologous DHAV-1 strain DRL-62 (group 1.1) and heterologous DHAV-1 strain D-20220906 (group 3), demonstrating full protection (100% survival) against the homologous strain DRL-62 (group 1.1) challenge and partial protection (40% survival) against the heterologous strain D-20220906 (group 3). At the same time, ducklings with MDA received an additional vaccination at 1 day of age and were challenged at 2 DPV. All ducklings exhibited 100% survival, regardless of the virus strain, and none displayed any clinical symptoms up to 7 days post-challenge (DPC) (Table 4).

### 3.4. Protective Efficacy of DHAV-3 Vaccine (Group 2)

The efficacy of the DHAV-3 vaccine strain AP-04203P100 (group 2) was evaluated in two experiments (experiment 2 and 4). In the first experiment (experiment 2), MDA-free ducklings were vaccinated and subsequently challenged on 2 and 4 DPV with either the homologous DHAV-3 virulent strain AP-04203 (group 2) or the heterologous DHAV-3 variant field strain D-20220615 (group 1). By 2 DPV, complete protection against the homologous DHAV-3 strain AP-04203 (group 2) was achieved, with all ducklings surviving the challenge and remaining symptom-free throughout the study period (Table 5). Similarly, the results for the heterologous DHAV-3 strain D-20220615 (group 1) challenge mirrored those of the homologous challenge, with complete protection observed from 2 DPV onwards.

In the second experiment (experiment 4), the protective efficacy of the DHAV-3 vaccine strain AP-04203P100 (group 2) was evaluated in ducklings with MDA. The initial antibody titers in 1-day-old ducklings were recorded at 2.8 ± 0.4 log2 against the homologous DHAV-3 virulent strain AP-04203 (group 2). Ducklings with MDA were vaccinated on the first day of life and subsequently challenged with both homologous DHAV-3 strain AP-04203 (group 2) and heterologous DHAV-3 strain D-20220615 (group 1) at 2 DPV. Remarkably, all vaccinated and non-vaccinated ducklings survived until the end of the experiment, demonstrating complete protection, regardless of vaccination status (Table 6).

## 4. Discussion

DHAV infections, particularly those caused by DHAV-1 and DHAV-3, present severe threats to duckling populations, leading to acute hepatitis, high mortality rates, and significant economic losses globally. The rapid spread and virulence of DHAV strains underscore the urgent need for effective vaccines to control outbreaks. Although live attenuated vaccines for DHAV-1 and DHAV-3 have been extensively implemented, genetic variations among circulating field isolates diminish the cross-protective efficacy of these vaccines [3,4]. This study aimed to evaluate the cross-protection afforded by current DHAV vaccines, specifically focusing on the protective effectiveness of DHAV-1 vaccines (group 1.1) against DHAV-1 variant field strain (group 3), and the cross-protection of DHAV-3 vaccines (group 2) against DHAV-3 variant field strain (group 1). These assessments underscore the limitations of existing vaccines and offer insights into the need for improved vaccine designs that address cross-genotypic variability in the prevention and control of DHAV.

Live attenuated DHAV vaccines remain a primary method for inducing immune protection and controlling duck hepatitis, with previous studies confirming their effectiveness against genotype-matched homologous field strains of both DHAV-1 and DHAV-3 [4,17]. However, our results indicate that these vaccines are less effective against genetically divergent field strains, notably the increasingly prevalent DHAV-1 strain (group 3) [14]. This finding is consistent with prior research indicating that the current vaccines, particularly DHAV-1 vaccines (group 1), exhibit reduced capacity to neutralize divergent strains [14]. The limited cross-protection observed in DHAV-1 may be due to its weaker neutralizing effect against heterologous genotypes. Our study also demonstrates that, despite extensive use of group 1 and 2 vaccines in China, emergent DHAV-1 group 3 and 4 field strains persist among vaccinated ducks [14,16]. Moreover, group 4 strains continue to spread among ducks vaccinated with group 1 vaccines in Egypt [15,16,25]. This trend emphasizes the need for genotype-matched vaccines that can effectively counter the genetic variability observed in DHAV-1, ensuring robust defense against evolving field strains.

Maternal-derived antibodies (MDAs) are critical for providing early immunity in ducklings, offering an initial line of defense until active immunity can be induced through vaccination [4,8]. However, our findings indicate that while MDA offers some protection, its effectiveness against genetically divergent DHAV-1 strains appears limited. This limitation may stem from the genetic differences between MDA derived from DHAV-1 group 1-vaccinated breeders and the group 3 variant field strain, which may not be adequately recognized or neutralized by the MDA. Moreover, our study also reveals that immunizing ducklings carrying MDA significantly enhances their protective response, achieving full immunity against genetically divergent DHAV-1 strains (Table 4). This suggests that, while MDA alone may not fully protect against variant field strains, combining passive immunity with additional active immunization can provide robust and long-lasting immunity across different genotypes. These findings support earlier research indicating that combining passive immunization with live attenuated vaccines can increase antibody levels and enhance protective efficacy [26], highlighting the potential benefits of combining MDA with live vaccines for more comprehensive immunity in ducklings. Similarly, because of the priming benefit of live vaccines in MDA-existing birds, vaccination strategies against ND in young chicks were conducted by combining MDA with live attenuated vaccines [9]. Additionally, previous studies have also shown that IgY-based passive immunization in ducks provides a valuable approach for preventing and treating infections, particularly those caused by DHAV [8,27]. Together, these strategies underscore the potential of MDA and live vaccine immunization as a comprehensive approach to enhancing immunity in ducklings [28]. To effectively counter the emergence of variant DHAV strains, further research should also prioritize the development of optimized vaccination and treatment protocols that ensure continuous, high-level immunity across genetic variants.

Mutations in the DHAV-1 genome, particularly within the VP1 protein, significantly impact viral virulence and immune-escape mechanisms, posing challenges to current vaccine efficacy [4,29]. The VP1 protein is essential for viral attachment to host cells, and contains the primary neutralization epitope; therefore, its variability can modify antigenicity and affect antibody recognition across DHAV-1 strains [30,31,32]. In our study, the DHAV-1 variant field strain D-20220906 (group 3) exhibited eight amino acid substitutions and one deletion in VP1 compared to the DHAV-1 vaccine strain HSB-P100 (group 1.1), with mutations T180A, S181L, R183Q, I189V, R231M, H219Y, and a deletion at position 187 localized within hypervariable regions HVR-2 (180–193) and HVR-3 (213–219). This mutation pattern, especially within HVR regions, may facilitate immune escape, as evidenced by the reduced neutralization efficacy of antibodies from the vaccine strain HSB-P100 (group 1.1) against variant field strain D-20220906 (group 3), with duckling challenge results further supporting this hypothesis. Similar findings from previous studies suggest that mutations in the HVR region could promote immune evasion through antigenic shifts [33,34]. Additionally, under vaccine-induced selection pressure, mutations in DHAV HVR regions, including S182P, G/E184K, N186K, and V187D, have been previously observed [16]. Though our findings differ in specific mutations from a previous study, variations in vaccine strains and environmental conditions could explain this divergence [35]. These results underscore the importance of investigating VP1 mutations to better understand immune-escape mechanisms and inform the development of more effective DHAV vaccines.

Despite the genetic diversity between vaccines and variant field strains, DHAV-3 vaccines demonstrated strong cross-protective efficacy. Both virus neutralization assays and in vivo experiments showed that ducks vaccinated with DHAV-3 vaccine strain AP-04203P100 (group 2) achieved complete protection against heterologous DHAV-3 variant field strain D-20220615 (group 1) as early as two days post-vaccination. Ducklings carrying MDA also showed effective protection against heterologous DHAV-3 variant field strains without requiring a booster at one day of age. These observations suggest that DHAV-3 may elicit a broader immune response, capable of cross-protecting against genetically distant genotypes (group 1 vs. group 2). For the mutations in VP1, compared with eight amino acid substitutions in VP1-HVR regions of DHAV-1 variant field strains, DHAV-3 variant field strain D-20220615 (group 1) exhibits sixteen amino acid substitutions compared to DHAV-3 vaccine strain AP-04203P100 (group 2), and nine amino acid substitutions in the VP1-HVR region. This may be influenced by the evolutionary characteristics of viruses. DHAV-3 has a faster-evolving power than DHAV-1 by three times [32], and many amino acid substitutions between different DHAV-3 strains were detected. However, the correlation between amino acid substitutions and the antigenicity of DHAV-3 was not fully characterized very well.

These findings also suggest potential differences in immunogenicity between DHAV-1 and DHAV-3, as DHAV-3’s broader cross-protective effect may originate from inherent differences in its immunogenic profile [4,14]. Such distinctions warrant further exploration of the mechanisms governing immune responses and immune escape among DHAV variants.

## 5. Conclusions

In summary, the observed differences in cross-protection between the DHAV-1 and DHAV-3 vaccines underscore the necessity for continuous monitoring of circulating DHAV strains and adaptable vaccine strategies. Additionally, combining passive immunity with active immunization can provide robust immunity across multiple DHAV-1 genotypes. The pronounced cross-protective efficacy of DHAV-3 vaccines against genetically diverse strains highlights the potential for further research into the immunogenic properties of DHAV-3, which could serve as a model for developing broader-spectrum vaccines. Future studies should concentrate on elucidating the immune mechanisms that underpin the broader protection offered by DHAV-3 and identifying specific genetic markers associated with immune escape in DHAV. As viral evolution continues to challenge existing vaccine formulations, the development of multivalent vaccines that target multiple genotypes, coupled with enhanced genomic surveillance, will be crucial for maintaining effective control over DHAV outbreaks in commercial duck populations.

## Figures and Tables

**Table 1:** Results of cross-neutralization tests for DHAV-1 strains.

Virus (Genotype)	Anti-Serum Against Different DHAV-1 Strains		
	DRL-62 (Group 1.1)	D-20220155 (Group 3)	D-20220906 (Group 3)
DRL-62 (group 1.1)	256	61	54
D-20220155 (group 3)	32	225	194
D-20220906 (group 3)	43	168	256

**Table 2:** Results of cross-neutralization tests for DHAV-3 strains.

Virus (Genotype)	Anti-Serum Against Different DHAV-3 Strains		
	D-20220615 (Group 1)	D-202210113 (Group 1)	AP04203 (Group 2)
D-20220615 (group 1)	134	116	108
D-202210113 (group 1)	118	128	104
AP04203 (group 2)	62	68	94

**Table 3:** Protective efficacy of the DHAV-1 vaccine (group 1.1) against variant virus.

Groups	Challenge Virus	Survival Rate (%)	
		2 DPV	4 DPV
Non-vaccination	DRL-62 (group 1.1)	0 (0/10)	10 (1/10)
Vaccination		60 (6/10)	100 (10/10)
Non-vaccination	D-20220906 (group 3)	0 (0/10)	0 (0/10)
Vaccination		40 (4/10)	60 (6/10)

**Table 4:** Protective efficacy of the DHAV-1 vaccine (group 1.1) against variant virus in ducklings with MDA (3.4 ± 0.4 log2).

Groups (MDA)	Challenge Virus	Survival Rate (%)
Non-vaccination	DRL-62 (group 1.1)	100 (10/10)
Vaccination		100 (10/10)
Non-vaccination	D-20220906 (group 3)	40 (4/10)
Vaccination		100 (10/10)

**Table 5:** Protective efficacy of the DHAV-3 vaccine (group 2) against variant virus.

Groups	Challenge Virus	Survival Rate (%)	
		2 DPV	4 DPV
Non-vaccination	AP-04203 (group 2)	20 (2/10)	40 (4/10)
Vaccination		100 (10/10)	100 (10/10)
Non-vaccination	D-20220615 (group 1)	0 (0/10)	0 (0/10)
Vaccination		100 (10/10)	100 (10/10)

**Table 6:** Protective efficacy of the DHAV-3 vaccine (group 2) against variant virus in ducklings with MDA (2.8 ± 0.4 log2).

Groups (MDA)	Challenge Virus	Survival Rate (%)
Non-vaccination	AP-04203 (group 2)	100 (10/10)
Vaccination		100 (10/10)
Non-vaccination	D-20220615 (group 1)	100 (10/10)
Vaccination		100 (10/10)

## Data Availability

The data presented in this study are available from the corresponding author on reasonable request.
